# Prevalence of vestibulo-ocular reflex dysfunction in people with neurological disorders: a systematic review and meta-analysis

**DOI:** 10.1007/s00415-026-13619-1

**Published:** 2026-01-21

**Authors:** Nicola Ferri, Michael C. Schubert, Elisa Ravizzotti, Alessandro Bracci, Giacomo Metta Franceschelli, Diego Piatti, Paolo Pillastrini, Andrea Turolla, Marco Tramontano

**Affiliations:** 1 Translational Rehabilitation Sciences Group, Department of Biomedical and Neuromotor Sciences (DIBINEM), Alma Mater Studiorum University of Bologna, 40138 Bologna, Italy; 2 Department of Otolaryngology-Head and Neck Surgery, The Johns Hopkins School of Medicine, Baltimore, MD USA; 3 Department of Physical Medicine and Rehabilitation, The Johns Hopkins School of Medicine, Baltimore, MD USA; 4 Department of Neuroscience, Rehabilitation, Ophthalmology, Genetics and Maternal Child Health, University of Genoa, Genoa, Italy; 5 Department for Life Quality Studies (QUVI), University of Bologna, Rimini, Italy; 6 Rehabilitation Unit, Maggiore Hospital AUSL Bologna, Bologna, Italy; 7 Laboratory of Neuromotor Physiology, Santa Lucia Foundation, Scientific Institute for Research and Health Care, Rome, Italy; 8 IRCCS Azienda Ospedaliero-Universitaria di Bologna, Bologna, Italy

**Keywords:** Central nervous system diseases, Head impulse test, Dizziness, Vertigo, Meta-analysis

## Abstract

**Background:**

Neurological disorders, a leading cause of global disability, often cause debilitating dizziness and imbalance. While subjective symptoms are well-documented, the actual prevalence of vestibulo-ocular reflex (VOR) dysfunction in patients with central nervous system (CNS) damage remains unclear due to inconsistent primary studies. This research aims to determine the prevalence of VOR gain dysfunction, as measured by the video Head Impulse Test (vHIT), across neurological disorders.

**Methods:**

Our systematic review searched MEDLINE, CENTRAL, CINAHL, Scopus, ClinicalTrials.gov, and the WHO ICTRP for original articles from 2009 to September 2025. The JBI Checklist for prevalence studies was used to assess the methodological quality, and descriptive analyses were performed, followed by a meta-analysis of proportions using a random-intercept logistic regression model.

**Results:**

We included 48 studies, of which three reported on the same or overlapping samples. Thus, 45 unique studies (1604 participants, 792 females, mean age 56) were described. A meta-analysis of 33 studies (1129 participants) found an overall prevalence of vestibular dysfunction of 48% (95% CI 31–67%). Given the high heterogeneity, we performed subgroup analyses by condition. We found a pooled prevalence of 98% for CANVAS, 73% for ataxia, 44% for Parkinson’s disease, 59% for multiple sclerosis, 15% for traumatic brain injury, 5% for multiple system atrophy, and 77% for superficial siderosis.

**Conclusion:**

Isolated semicircular canal dysfunctions, as documented using vHIT, are prevalent in neurological disorders. Future research must elucidate their etiology and diagnostic potential, utilizing comprehensive vestibular assessments. Eventually, these findings should be translated into improved, evidence-based rehabilitation strategies.

**PROSPERO registration:**

CRD42024575542.

**Supplementary Information:**

The online version contains supplementary material available at 10.1007/s00415-026-13619-1.

## Background

Neurological disorders represent a paramount global health challenge, constituting the leading cause of disability-adjusted life years worldwide. Recent estimates indicate that these conditions affect up to 43.1% of the population, with stroke being the single most significant contributor to this burden [1]. Among the most common and debilitating symptoms experienced by this patient population are dizziness and postural instability [2]. These symptoms not only severely impact quality of life and functional independence but also significantly increase the risk of falls, leading to further morbidity and mortality [3].

The vestibular system is a fundamental part of sensorimotor function, playing a key role in maintaining balance, ensuring gaze stability during head movements, providing spatial orientation, and even contributing to cognitive processes such as navigation and memory [4, 5]. This complex system, comprising the peripheral organs in the inner ear and their central pathways, works in concert with the visual and proprioceptive systems. Sensory information from the semicircular canals and otolithic organs is integrated and processed within the central nervous system (CNS) to generate appropriate motor responses, thereby ensuring stability during everyday activities and facilitating compensation following vestibular loss [4, 6]. Consequently, CNS damage from a neurological event can disrupt this intricate network, leading to central vestibular dysfunction that manifests as dizziness and imbalance, even in the absence of direct peripheral vestibular damage [7].

The clinical assessment of vestibular function has been revolutionized by the advent of the video head impulse test (vHIT). This objective, non-invasive tool allows precise quantification of the vestibulo-ocular reflex (VOR) gain, which is the ratio of eye velocity to head velocity, providing a direct functional measure of each of the six semicircular canals [8]. The vHIT has proven to be a safe, reliable, and well-tolerated tool, leading to its widespread adoption across diverse clinical settings for diagnostic purposes, differential diagnosis, and monitoring of vestibular compensation [9–11]. Its application has been particularly valuable in neurology, where it helps distinguish between peripheral and central causes of vertigo [12].

While a vast body of literature has established the high prevalence of self-reported dizziness and clinical balance deficits in neurological populations such as stroke, multiple sclerosis, and Parkinson’s disease [2], the objective characterization of the underlying vestibular impairment remains less explored. The vHIT provides a unique opportunity to quantify the specific functional integrity of the VOR in these patients. To date, numerous primary studies have investigated VOR gain using vHIT in various neurological cohorts, reporting heterogeneous prevalence rates of dysfunction. However, a comprehensive synthesis of this evidence is lacking.

Therefore, the present systematic review with meta-analysis aims to consolidate the existing evidence to determine the point prevalence of VOR gain dysfunction, as measured by the vHIT, in people with a range of neurological disorders. By providing a pooled estimate of prevalence, this work could clarify the extent of vestibular physiology involvement in CNS pathologies, inform clinical assessment protocols, and highlight potential targets for vestibular physical therapy.

## Methods

This systematic review and meta-analysis is reported in adherence with the Preferred Reporting Items for Systematic Reviews and Meta-analyses (PRISMA) [13] and the Meta-analysis of Observational Studies in Epidemiology (MOOSE) [14], given that the PRISMA extension for systematic reviews of prevalence (PRISMA-Prev) is still under development. This study protocol was prospectively registered on PROSPERO (https://www.crd.york.ac.uk/PROSPERO/view/CRD42024575542).

### Search strategy and selection criteria

We searched MEDLINE, CENTRAL, CINAHL, and Scopus from 2009 to September 2025. ClinicalTrials.gov and the World Health Organization ICTRP were also scanned for gray literature. Additionally, we reviewed the reference lists of the included studies and consulted experts in the field to identify any current, eligible articles. No language restrictions were applied. The publication date limit from 2009 was used, given the first paper ever published on the vHIT [15].

We identified keywords related to the research question, then combined free text and MeSH terms using Boolean operators. The search strategy was supervised by our librarians following the PRESS (Peer Review of Electronic Search Strategies) guideline statement [16]. A complete search strategy for each database is reported in the eMethods1 (Supplement 1).

Two authors independently screened the titles and abstracts, and then the full texts; any discrepancies were resolved through discussion. Original articles were included only when they reported the prevalence of VOR gain dysfunction, as measured by the vHIT, in populations with CNS disorders. Although prevalence studies are typically expected to employ cross-sectional designs, we included all study designs that reported our data of interest, excluding abstracts or conference papers. We also excluded articles that met the above criteria but had relevant confounding factors, such as participants with CNS disorders who were also associated with unilateral vestibular hypofunction, as in Micarelli et al. [17]. Moreover, we excluded any emergency setting, as the acute phase of a pathology can be confusing due to possible misdiagnosis, transient vestibular hyper- or hypo-function, or recovery.

### Data analysis

Data were extracted independently by two authors using a predefined and piloted spreadsheet. Any conflicts were solved by consensus. In particular, the following variables were collected: first author, publication year, title, country, study design, sample size, mean age, sex, diagnosis, setting, vHIT name, normative cutoff, mean VOR gain for each semicircular canal, and prevalence of participants with at least one dysfunctional semicircular canal.

Vestibular dysfunction was operationally defined as the presence of at least one semicircular canal with an abnormal VOR gain, as assessed using the vHIT. The definition of abnormal gain followed the cutoff values adopted in each primary study, which were based on manufacturer normative data or clinical database previously published reference values. Variability in vHIT protocols, devices (e.g., Otometrics, EyeSeeCam, SLMED), and gain cutoff thresholds was accepted, in line with the prevalence-focused nature of this review. Importantly, even within the same vHIT device, distinct normative cutoffs were commonly applied for horizontal and vertical semicircular canals, reflecting known physiological and technical differences across semicircular canal planes. For example, when using Otometrics systems, normal VOR gain ranges were often defined as 0.76–1.29 for vertical semicircular canals and 0.80–1.29 for horizontal semicircular canals, whereas studies using EyeSeeCam frequently adopted a more conservative threshold (e.g., VOR gain < 0.7) to define semicircular canal dysfunction.

In some studies, vestibular integrity was additionally inferred from the absence of compensatory saccades during head impulses; however, to ensure methodological consistency across pooled prevalence estimates, only VOR gain–based definitions of dysfunction were considered eligible for quantitative analysis.

The JBI Checklist for prevalence studies [18] was used to evaluate the methodological quality of eligible articles. This tool consists of 9 questions with 4 possible responses (Yes, No, Unclear, Not applicable). It investigates the appropriateness of the sample, the reporting of participants and setting, the methods for measuring condition prevalence, and the data analysis details. We did not create an overall score to avoid distorting the scale’s construct, losing data, or making the unproven assumption that items are equally weighted. The complete quality assessment was independently performed by two researchers, with any conflicts solved by a third reviewer.

We conducted descriptive analyses using means and standard deviations (or medians and interquartile ranges) for continuous outcomes, and counts and percentages for categorical outcomes. For each study, we calculated the point prevalence of vestibular dysfunction by dividing the sample into functional (people with no dysfunctional semicircular canals) and dysfunctional (people with ≥ 1 dysfunctional semicircular canals) groups.

The analysis was conducted using the PERSyst-MA tool version 1.0, an online application for R package meta, version 7.0-0. A meta-analysis of proportions was performed using a random-effects model. Heterogeneity was measured using maximum-likelihood as the variance estimator. The meta-analysis used a random intercept logistic regression model, with the logit transformation applied to the proportions. A continuity correction of 0.5 was applied in studies with zero cell frequencies.

Subgroup analyses were performed, and clinical and methodological factors were examined to identify heterogeneity. When multiple studies reported on the same sample or had partial data overlap, the larger sample was selected and analyzed to prevent double-counting. If a single article presented data from two different populations, the article was split into two samples (e.g., Kim 2022 into Kim 2022a and Kim 2022b) for subgroup analyses.

We did not perform a funnel plot to investigate publication bias because the subgroup meta-analyses did not include the minimum recommended studies (i.e., n < 10).

## Results

**Fig. 1:**
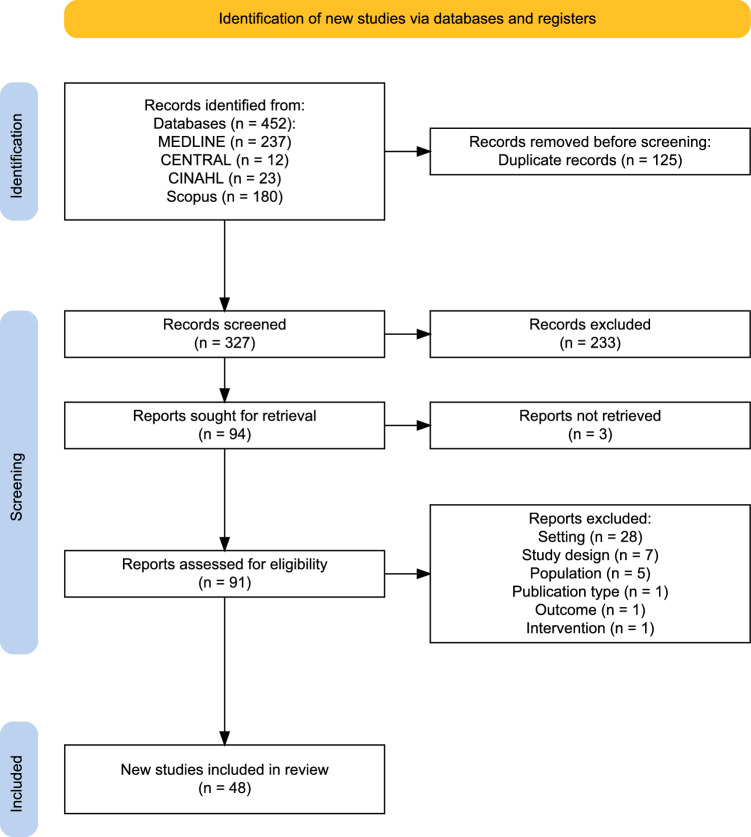
PRISMA flowchart

A total of 452 records were retrieved through our search strategy; after removing 125 duplicates, 327 were screened for inclusion criteria. Finally, we included 48 studies [19–66] in the review (Fig. 1). Two studies [32, 34] refers to the same sample; thus, we considered the data only once in the statistical analyses. Three studies [37, 61, 65] presented data on overlapping samples, thus the largest sample [61] was retained for meta-analyses purposes, to avoid double-counting. A list of excluded full-text studies, along with their rationales, is provided in eMethods2 (Supplement 1).

Overall, the 45 unique studies included 1604 participants (792 females, mean age 56) and examined a range of neurological diseases. The full characteristics of unique samples are reported in Table 1.

**Table 1:** Studies characteristics

Article	Country	Sample size	Diagnosis	Age, years	Sex, female	vHIT device
Alshehri et al. [19]	USA	56	Concussion	23.2	31	EyeSeeCam
Anagnostou et al. [20]	Greece	7	KD	50.3	0	EyeSeeCam
Ariello et al. [59]	USA	20	SCA	74.5	10	Otometrics
Aydin Canturk et al. [22]	Turkey	35	MS	38.5	22	Otometrics
Berkiten et al. [21]	Turkey	40	PD	63.2	16	Otometrics
Borsche et al. [23]	Germany	20	CANVAS	67.8	6	EyeSeeCam
Bosmans et al. [24]	Belgium	50	MCI, AD	74.7	22	Otometrics
Bremova et al. [25]	Germany	8	NPC	27.3	2	EyeSeeCam
Choi et al. [26]	South Korea	26	SCA	52.0	17	SLMED
Dankova et al. [27]	Czech Republic	32	SCA, MSA-C, ILOCA	60.7	NR	Otometrics
Demir et al. [28]	Turkey	25	Epilepsy	32.4	8	EyeSeeCam
Egilmez et al. [29]	Turkey	53	MS	35.6	45	EyeSeeCam
Elyoseph et al. [30]	Israel	35	MJD	57.0	24	Otometrics
Feller et al. [63]	USA	30	TBI	36.8	16	Otometrics
Fernandez-Rueda et al. [31]	Spain	7	CANVAS	56.0	6	Otometrics
Ferri et al. [60]	Italy	35	PD	69.9	11	Otometrics
Ferri et al. [62]	Italy	21	TBI	48.0	3	Otometrics
Grove et al. [33]	USA	12	MS	55.5	NR	Otometrics
Grove et al. [34]	USA	37	MS	53.4	28	Otometrics
Hawkins et al. [35]	Australia	40	PD	69.6	13	Otometrics
Heravian Shandiz et al. [36]	Iran	26	MS	36.4	20	NR
Hong et al. [37]	South Korea	133	PD	68.0	74	SLMED
Hougaard et al. [38]	Denmark	8	MELAS	49.6	6	EyeSeeCam
Kim et al. [39]	South Korea	53	MSA, PD	67.1	27	SLMED
Kim et al. [40]	South Korea	33	SCA	45.9	16	Otometrics
Kim et al. [65]	South Korea	138	PD	70.0	67	SLMED
Kim et al. [61]	South Korea	151	PD	68.0	74	SLMED
Le et al. [41]	USA	25	TBI	52.0	0	Otometrics
Lee et al. [42]	South Korea	12	SCA	56.0	6	SLMED
Lemos et al. [43]	Portugal	38	SCA	49.8	24	EyeSeeCam
Luis et al. [44]	Portugal	32	FA, SCA	45.4	15	EyeSeeCam
Lv et al. [45]	China	63	PD	65.7	26	Otometrics
Millar et al. [46]	USA	19	SCA	61.0	10	Otometrics
Oron et al. [47]	Israel	7	SUSAC	36.9	4	EyeSeeCam
Pavlovic et al. [48]	Croatia	29	MS	33.7	14	EyeSeeCam
Scarpa et al. [49]	Italy	31	MSA, PD	64.3	12	Otometrics
Sonkaya et al. [66]	Turkey	40	PD	61.1	17	EyeSeeCam
Surmeli et al. [51]	Turkey	9	Epilepsy	37.1	5	EyeSeeCam
Surmeli et al. [50]	Turkey	27	MS	39.3	21	EyeSeeCam
Takeda et al. [52]	Japan	10	SS	69.2	2	EyeSeeCam
Taylor et al. [53]	New Zealand	99	TBI	49.0	59	Otometrics
Tramontano et al. [54]	Italy	36	Stroke	55.1	11	Otometrics
Tramontano et al. [64]	Italy	27	MS	47.9	18	Otometrics
Wagner et al. [56]	Switzerland	5	SS	61.4	1	Otometrics
Wagner et al. [55]	USA	40	MS	53.9	31	Otometrics
Woo et al. [57]	South Korea	140	PD	68.0	71	SLMED
Yacovino et al. [58]	Argentina	5	CANVAS	73.0	3	EyeSeeCam

*KD* Kennedy disease, *SCA* spinocerebellar ataxia, *MS* multiple sclerosis, *PD* Parkinson’s disease, *CANVAS* cerebellar ataxia with neuropathy and vestibular areflexia syndrome, *MCI* mild cognitive impairment, *AD* Alzheimer’s disease, *NPC* Niemann–Pick disease type C, *MSA-C* multiple system atrophy-cerebellar subtype, *ILOCA* idiopathic late-onset cerebellar ataxia, *MJD* Machado-Joseph disease (i.e., SCA type 3), *TBI* traumatic brain injury, *MELAS* mitochondrial encephalopathy, lactic acidosis, and stroke-like episodes, *FA* Friedreich’s ataxia, *SUSAC* also known as retinocochleocerebral vasculopathy, *MSA* multiple system atrophy, *SS* superficial siderosis, *NR* not reported

**Fig. 2:**
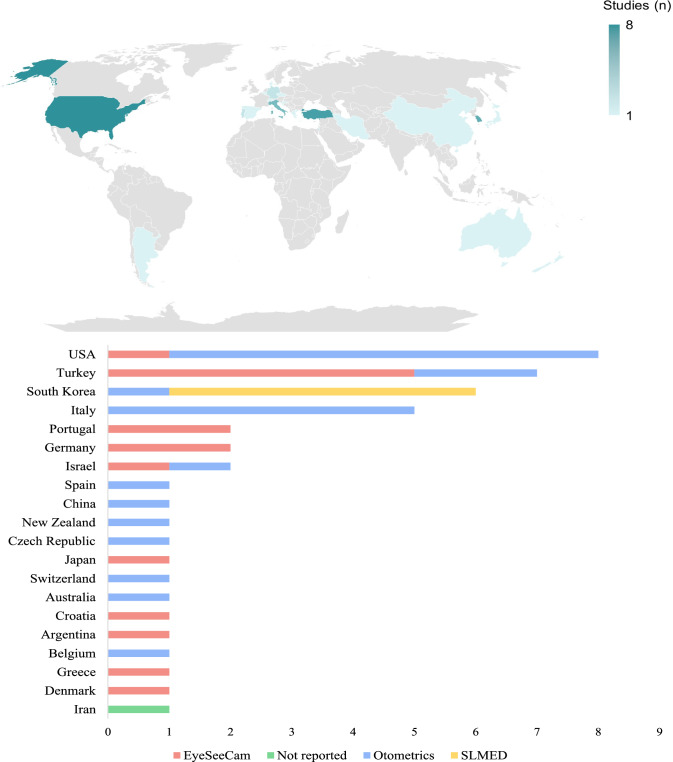
vHIT prevalence by country

Included studies adopted vHIT devices from Otometrics (51%), EyeSeeCam (36%), SLMED (11%), or did not report the manufacturer (2%). Figure 2 shows the geographic distribution of vHIT devices. The USA was the largest user of Otometrics (n = 7), South Korea was the only SLMED user among all included countries (n = 5), Italy was represented only by Otometrics (n = 5), and both Otometrics and EyeSeeCam were present on all continents.

### Assessment of quality (JBI checklist for prevalence studies)

**Fig. 3:**
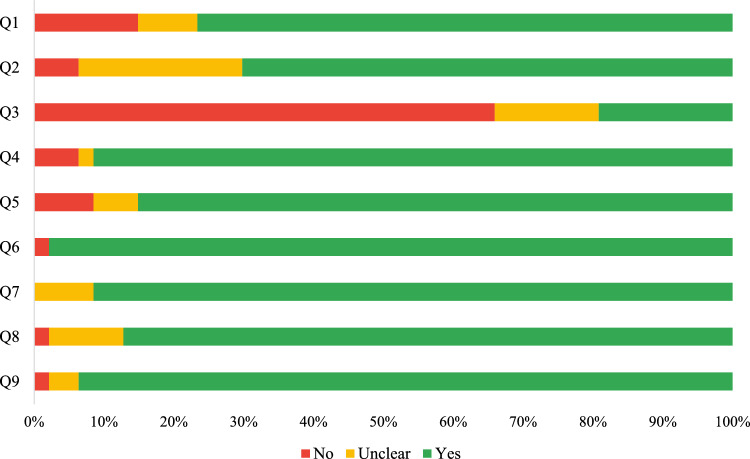
Quality assessment

Methodological quality was overall sound (Fig. 3), with a single concern regarding sample size adequacy (Q3 item) in 31 studies (66%). Complete assessments of all included studies are reported in eMethods3 (Supplement 1).

### Prevalence of vestibular dysfunctions

**Fig. 4:**
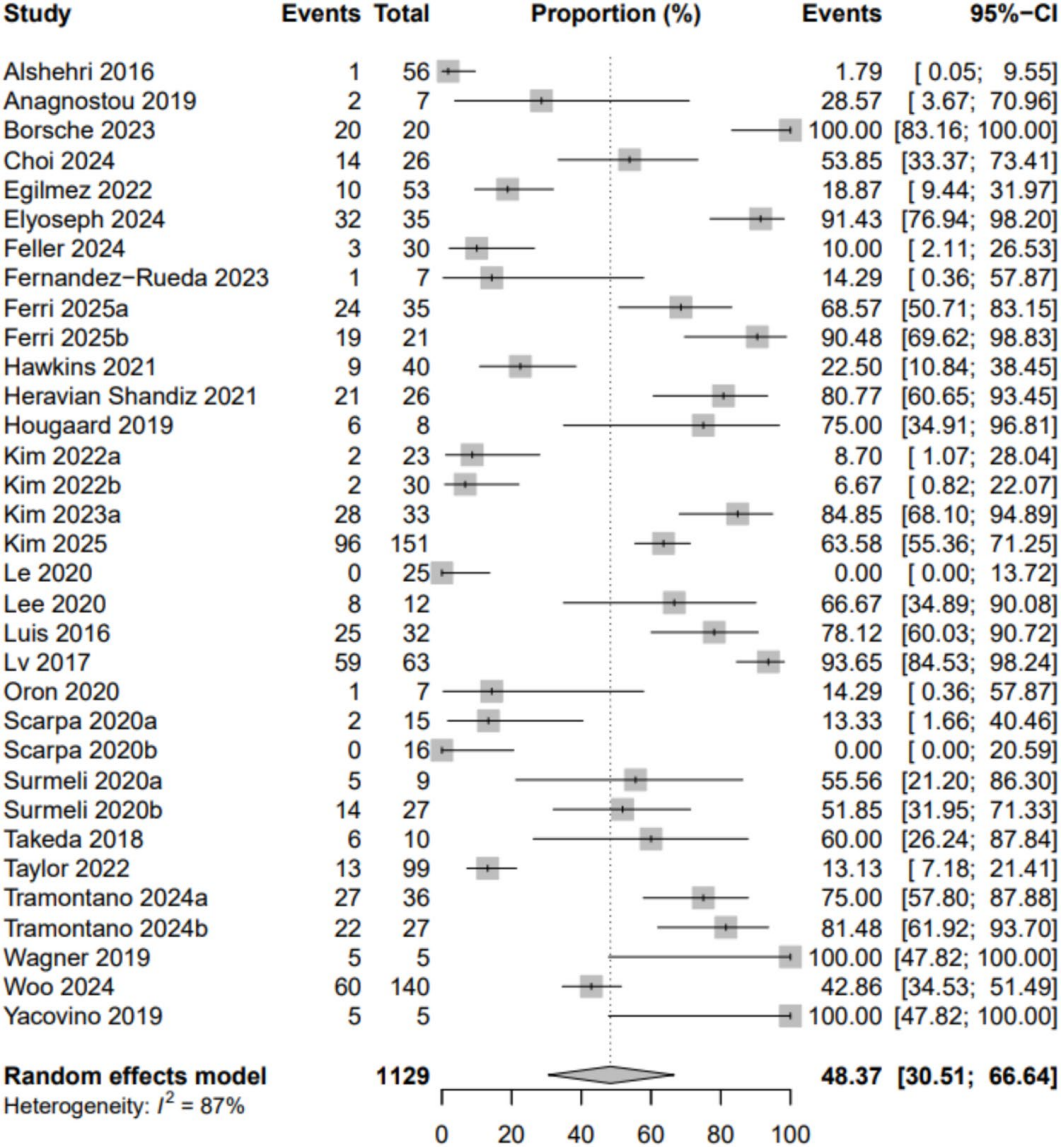
Meta-analysis (33 studies, 1129 participants)

In the overall meta-analysis (Fig. 4), aggregating all unique studies reporting point estimates, the pooled prevalence of vestibular dysfunction in people with CNS disorders is 48% [95% CI 31–67]. The heterogeneity identified in this global analysis is significant (Table 2).

**Table 2:** Heterogeneity assessment

Quantifying heterogeneity:			
Tau^2^ = 4.3638	Tau = 2.0890	I^2^ = 87.4%	H = 2.81

Test of heterogeneity:	Q	d.f.	p-value
Wald	253.18	32	< 0.0001
LRT	529.66	32	< 0.0001

Thus, to investigate heterogeneity, subgroup analyses were conducted by CNS condition, and further methodological and clinical assessments were performed when necessary.

### Cerebellar ataxia, neuropathy, vestibular areflexia syndrome (CANVAS)

**Fig. 5:**
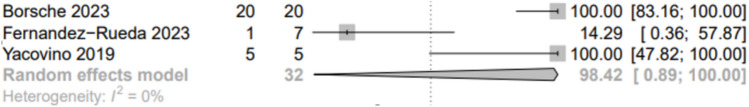
CANVAS subgroup

Three studies [23, 31, 58] (23 participants) investigated people with CANVAS and found a 98% pooled prevalence of vestibular dysfunction, with no substantial heterogeneity (Fig. 5).

### Spinocerebellar ataxia (SCA)

**Fig. 6:**
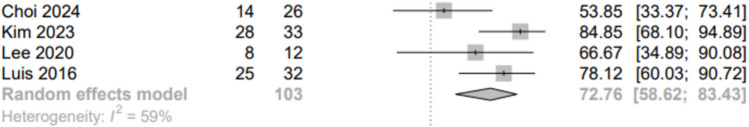
Spinocerebellar ataxia subgroup

Four studies [26, 40, 42, 44] investigated vestibular dysfunctions in people with ataxia; the pooled prevalence was 73%, with moderate heterogeneity (Fig. 6).

Another four studies [27, 43, 46, 59] did not report a direct prevalence estimate for meta-analysis, but still provided valuable information. In particular, Ariello et al. [59] found an abnormal VOR gain in 45% of horizontal semicircular canals, 75% of anterior semicircular canals, and 95% of posterior semicircular canals in a sample of 20 adults (10 females, mean age 74.5). Moreover, the mean VOR gain was dysfunctional (< 0.8) in all vertical semicircular canals. Dankova et al. [27] analyzed 32 participants (mean age 60.7); they found an abnormal mean VOR gain in a subgroup of 12 participants (38%) for both vertical and horizontal canals. Lemos et al. [43] found significantly lower VOR gains in a sample of 38 people (24 females, mean age 49.8) than in healthy controls. Millar et al. [46] investigated a sample of 19 people (10 females, mean age 61). They found a significant prevalence of dysfunctional semicircular canals, in particular 58% for left horizontal, 68% for right horizontal, 37% for left anterior, 32% for right anterior, 11% for left posterior, 37% for right posterior canals.

### Parkinson’s disease (PD)

**Fig. 7:**
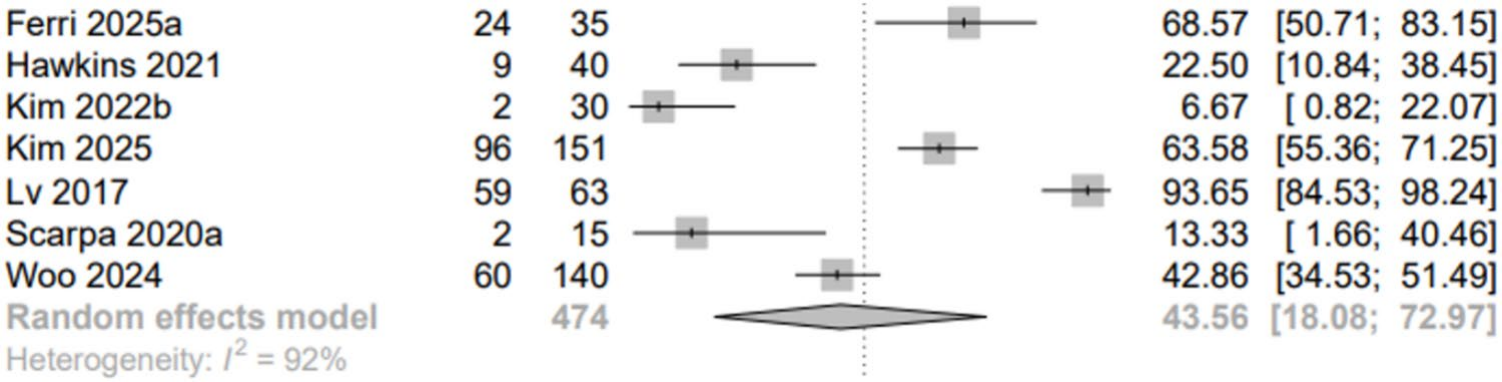
Parkinson’s disease subgroup

PD was the most prevalent condition in this review, with the largest sample size in the studies (Table 1). Seven studies [35, 39, 45, 49, 57, 60, 61] were pooled, yielding a prevalence of 44% for vestibular dysfunction in people with PD (Fig. 7). The heterogeneity was still high.

Berkiten et al. [21] investigated 40 people with PD (16 females, mean age 63.2) and found an abnormal mean VOR gain for left anterior (0.57) and right posterior (0.64) canals. Sonkaya et al. [66] analyzed 40 people with PD (17 females, mean age 61.1), and the mean VOR gain for the six canals was within the functional range.

### Multiple sclerosis (MS)

**Fig. 8:**
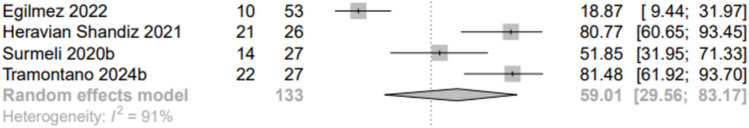
Multiple sclerosis subgroup

Four studies investigating people with MS had a pooled prevalence of 59% with substantial heterogeneity across estimates (Fig. 8). Two studies [29, 50] found lower prevalence, but they investigated or reported only horizontal canals VOR gains. Including only studies with complete vHIT assessment yields a prevalence of 81% [95% CI 68–90%] and no substantial heterogeneity.

Aydin Canturk et al. [22] studied 35 people with MS (22 females, mean age 38.5), finding a low mean VOR gain (0.6) for the left anterior canal. Grove et al. [33] investigated 12 people with MS (mean age 55.5); they found that moderate MS shows a lower lateral semicircular canal VOR gain bilaterally than mild MS, indicating that MS-related disability was associated with VOR gain. The same research group analyzed 37 people with MS [34] (28 females, mean age 53.4) and found that the mean VOR gains across most semicircular canals were worse and more dysfunctional as the disease stage worsened. Pavlović et al. [48] investigated 29 people with MS (14 females, mean age 33.7), resulting in 16/58 ears being dysfunctional.

### Traumatic brain injury (TBI)

**Fig. 9:**
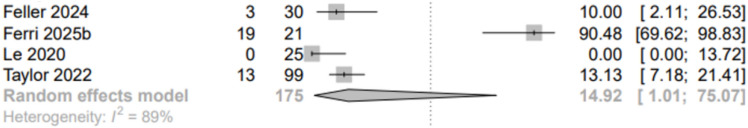
Traumatic brain injury subgroup

Four studies [41, 53, 62, 63] analyzing people with TBI were pooled, yielding a prevalence of 15%, with high heterogeneity (Fig. 9). Some methodological and clinical differences could explain the latter: an analysis of only horizontal canals that could have underestimated the prevalence [63], a more severe TBI population that could be correlated with a higher prevalence [62], and normal VOR gains yet with a high prevalence of corrective saccades [41].

Only one study [19] investigated mean VOR gains in people with concussion and reported a point prevalence of 2% [95% CI 0.1–10].

### Multiple system atrophy (MSA)

**Fig. 10:**
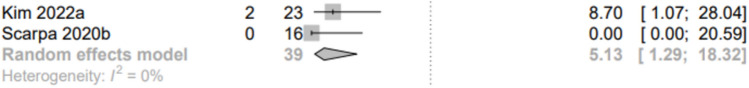
Multiple system atrophy subgroup

Two studies analyzed people with MSA; the pooled prevalence is 5%, with no substantial heterogeneity (Fig. 10).

### Stroke

Only one study [54] investigated people with stroke and found a prevalence of vestibular dysfunction of 75% (95% CI 58–88%).

### Superficial siderosis

**Fig. 11:**
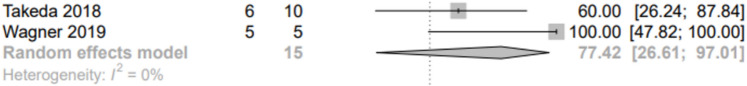
Superficial siderosis subgroup

Two studies [52, 56] analyzed people with superficial siderosis and found a prevalence of 77% for vestibular dysfunction, with no substantial heterogeneity (Fig. 11).

### Other neurological conditions

Single studies found vestibular dysfunction prevalence also in people with KD (29%) [20], MJD (91%) [30], MELAS (75%) [38], SUSAC (14%) [47], and epilepsy (56%) [51]. Demir et al. [28] also investigated vestibular function with vHIT in people with epilepsy, finding mean VOR gains within the functional range. Finally, two studies examined the mean VOR gain in lateral canals in people with MCI and AD [24], and in people with NPC [25], reporting functional values.

## Discussion

This is the first study to systematically report and synthesize the available evidence on vHIT-derived VOR gain abnormalities in people with neurological disorders. Based on our findings, we are 95% confident that the true prevalence of VOR dysfunction in the entire population analyzed ranges from 30 to 66%.

Among CNS disorders, stroke has the major impact on society, accounting for an incidence of 11.9 million and a loss of 160.5 million daily-adjusted life years worldwide [67]. However, in our systematic review, we could find only one study [54] reporting VOR dysfunction in people with stroke. We argue that this research gap is most likely due to the focus on stroke diagnosis using vHIT during an acute vestibular syndrome, a context excluded from our study since it is an emergency setting with possible distortion of VOR gains related to the very acute phase of the disease.

SCA, PD, and MS were the most consistent populations analyzed in our meta-analyses, with a prevalence of vestibular dysfunction of 73%, 44%, and 59% respectively. This could mean that, although it is often reported as a specific isolated semicircular canal dysfunction, the etiology may be non-specific and involve integration centers within the brainstem, cerebellar pathways, and along the nerve pathways that mediate signals between the vestibular nuclei, the cerebellum, and other parts of the brain (such as the thalamus and cerebral cortex).

Nevertheless, this significant prevalence rate highlights the importance of systematic vestibular assessment for individuals with neurological disorders in clinical settings. In particular, it is now clear that VOR gain, which was usually considered of peripheral interest, is often altered in central neurological disorders as well. This could have a future role in early diagnosis for specific populations, especially for isolated non-vestibulo-specific dysfunctions, and in applying current rehabilitation strategies by focusing on sensorimotor integration with awareness of overall balance function.

### Limitations

Some limitations of this research should be acknowledged. First, the VOR gain was the only output of the vHIT used to identify vestibular dysfunction; this is standard in the literature, with the cutoffs chosen by each study based on the manufacturer’s normative data, previous referenced research, or a healthy control population. Therefore, we did not consider other parameters, such as the presence, velocity, and latency of any compensatory saccades, which are increasingly gaining attention in recent literature [55, 66]. Second, our population also includes conditions of a genetic and metabolic nature, provided they have a neurological impact and reported VOR gain measurements. This inevitably introduced heterogeneity, though it was managed through consistent subgroup analysis. Third, did not include studies using the suppression head impulse test (SHIMP paradigm) as this has not been adopted as universally as vHIT. However, recent studies suggest that even with a normal VOR gain, central vestibular dysfunction may still be present due to the absence of a physiological anticompensatory saccade during vHIT, a sign of central pathology that is assessed using the SHIMP paradigm [60, 68]. Consequently, our estimates of the prevalence of vestibular dysfunctions might be underestimated. Fourth, we were unable to investigate publication bias due to the limited sample size; however, our comprehensive peer-reviewed bibliographic research and recent vHIT technology give us confidence that we have captured most of the existing literature. Finally, assessing the impact of device type on detecting vestibular dysfunction was not feasible due to insufficient sample sizes in each group. Nevertheless, our adherence to the cutoffs used in primary studies supports the consistency of the data.

## Conclusion

This evidence synthesis has shown that isolated semicircular canal dysfunctions are highly prevalent in people with neurological disorders, although they are currently underestimated in research and clinically under-investigated. Future primary research should focus on the etiopathology of these dysfunctions, assess their potential role in early diagnosis, analyze participants using various vestibular tests and indicators, and, ultimately, translate this knowledge into real-world settings to improve the rehabilitation approach and overall treatment effectiveness.

## Supplementary Information

Below is the link to the electronic supplementary material.

Supplementary file1 (DOCX 81 KB)

## Data Availability

All data supporting the findings of this study are available within the paper and the Supplementary materials.
