# Supplementary material for: Prevalence of vestibulo-ocular reflex dysfunction in people with neurological disorders: a systematic review and meta-analysis
Source: J Neurol. 2026 Jan 21;273(2):91. doi: 10.1007/s00415-026-13619-1 (PMC12823702; doi:10.1007/s00415-026-13619-1)
Supplement: Supplementary file 1 — Supplementary file1 (DOCX 81 KB) [file 415_2026_13619_MOESM1_ESM.docx]

**Prevalence of vestibulo-ocular reflex dysfunction in people with neurological disorders:
A systematic review and meta-analysis**

**Supplementary material**

**eMethods 1.** Complete search strategy

*Pubmed/MEDLINE*

(("Stroke"[Title/Abstract] OR "Stroke"[MeSH Terms] OR "Multiple Sclerosis"[Title/Abstract] OR "Multiple Sclerosis"[MeSH Terms] OR "Parkinson"[Title/Abstract] OR "parkinson disease"[MeSH Terms] OR "traumatic brain injur*"[Title/Abstract] OR "brain injuries, traumatic"[MeSH Terms] OR "central nervous system"[Title/Abstract] OR "central nervous system diseases"[MeSH Terms]) AND ("video head impulse test"[Title/Abstract] OR "vHIT"[Title/Abstract] OR "head impulse test"[MeSH Terms])) AND (2009:3000/12/12[pdat])

*CENTRAL*

#1 (stroke):ti,ab,kw
#2 MeSH descriptor: [Stroke] explode all trees
#3 (multiple sclerosis):ti,ab,kw
#4 MeSH descriptor: [Multiple Sclerosis] explode all trees
#5 ("Parkinson"):ti,ab,kw
#6 MeSH descriptor: [Parkinson Disease] explode all trees
#7 ("traumatic brain injury"):ti,ab,kw
#8 MeSH descriptor: [Brain Injuries, Traumatic] explode all trees
#9 (central nervous system):ti,ab,kw
#10 MeSH descriptor: [Central Nervous System] explode all trees
#11 (video head impulse test):ti,ab,kw
#12 (vHIT):ti,ab,kw
#13 MeSH descriptor: [Head Impulse Test] explode all trees
#14 (#1 OR #2 OR #3 OR #4 OR #5 OR #6 OR #7 OR #8 OR #9 OR #10) AND (#11 OR #12 OR #13)

*CINAHL*

S1 stroke
S2 multiple sclerosis
S3 parkinson
S4 traumatic brain injury
S5 central nervous system
S6 video head impulse test
S7 vHIT
S8 (S1 OR S2 OR S3 OR S4 OR S5)
S9 S6 OR S7
S10 S8 AND S9
S11 S8 AND S9 (Limiters – Publication Date: 20090101-20250926

*Scopus*

( ( TITLE-ABS-KEY ( stroke ) ) OR ( TITLE-ABS-KEY ( multiple AND sclerosis ) ) OR ( TITLE-ABS-KEY ( parkinson ) ) OR ( TITLE-ABS-KEY ( traumatic AND brain AND injury ) ) OR ( TITLE-ABS-KEY ( central AND nervous AND system ) ) ) AND ( ( TITLE-ABS-KEY ( video AND head AND impulse AND test ) ) OR ( TITLE-ABS-KEY ( vhit ) ) ) AND PUBYEAR > 2008 AND PUBYEAR < 2025

**eMethods 2.** List of excluded full texts with reasons

*Wrong setting (n = 28)*

1. Lee JY, Kim CH, Park JS, Kim MB. Peripheral Vestibulopathy Presenting as Acute Vertigo and Spontaneous Nystagmus with Negative Video Head Impulse Test. Otolaryngol Head Neck Surg. 2019 May;160(5):894-901. doi: 10.1177/0194599818825458. Epub 2019 Jan 22. PMID: 30665325.
2. Tamás TL, Garai T, Király I, Mike A, Nagy C, Paukovics Á, Schmidt P, Szatmári F, Tompos T, Vadvári Á, Szirmai Á. Az akut vestibularis szindróma sürgősségi diagnosztikájával szerzett tapasztalatok [Emergency diagnosis of the acute vestibular syndrome]. Orv Hetil. 2017 Dec;158(51):2029-2040. Hungarian. doi: 10.1556/650.2017.30886. PMID: 29250967.
3. Bery AK, Chang TP. Positive horizontal-canal head impulse test is not a benign sign for acute vestibular syndrome with hearing loss. Front Neurol. 2022 Sep 26;13:941909. doi: 10.3389/fneur.2022.941909. PMID: 36226090; PMCID: PMC9549073.
4. Calic Z, Nham B, Bradshaw AP, Young AS, Bhaskar S, D'Souza M, Anderson CS, Cappelen-Smith C, Cordato D, Welgampola MS. Separating posterior-circulation stroke from vestibular neuritis with quantitative vestibular testing. Clin Neurophysiol. 2020 Aug;131(8):2047-2055. doi: 10.1016/j.clinph.2020.04.173. Epub 2020 Jun 8. PMID: 32600960.
5. Guler A, Karbek Akarca F, Eraslan C, Tarhan C, Bilgen C, Kirazli T, Celebisoy N. Clinical and video head impulse test in the diagnosis of posterior circulation stroke presenting as acute vestibular syndrome in the emergency department. J Vestib Res. 2017;27(4):233-242. doi: 10.3233/VES-170620. PMID: 29081427.
6. Ha SH, Lee DK, Park G, Kim BJ, Chang JY, Kang DW, Kwon SU, Kim JS, Park HJ, Lee EJ. Prospective analysis of video head impulse tests in patients with acute posterior circulation stroke. Front Neurol. 2023 Sep 22;14:1256826. doi: 10.3389/fneur.2023.1256826. PMID: 37808489; PMCID: PMC10557255.
7. Kim HS, Oh EH, Kim JY, Choi SY, Choi KD, Choi JH. Discordant vestibulo-ocular reflex function according to the frequency and mode of stimulation. J Neurol. 2022 Sep;269(9):4742-4752. doi: 10.1007/s00415-022-11105-y. Epub 2022 Apr 8. PMID: 35394171.
8. Kim SH, Lee SU, Cho BH, Cho KH, Yu S, Kim BJ, Kim JS. Analyses of Head-Impulse Tests in Patients With Posterior Circulation Stroke and Vestibular Neuritis. Neurology. 2023 Jun 6;100(23):e2374-e2385. doi: 10.1212/WNL.0000000000207299. Epub 2023 Apr 19. PMID: 37076307; PMCID: PMC10256120.
9. Korda A, Wimmer W, Wyss T, Michailidou E, Zamaro E, Wagner F, Caversaccio MD, Mantokoudis G. Artificial intelligence for early stroke diagnosis in acute vestibular syndrome. Front Neurol. 2022 Sep 8;13:919777. doi: 10.3389/fneur.2022.919777. PMID: 36158956; PMCID: PMC9492879.
10. Korda A, Wimmer W, Zamaro E, Wagner F, Sauter TC, Caversaccio MD, Mantokoudis G. Videooculography "HINTS" in Acute Vestibular Syndrome: A Prospective Study. Front Neurol. 2022 Jul 12;13:920357. doi: 10.3389/fneur.2022.920357. PMID: 35903121; PMCID: PMC9314570.
11. Lee SH, Kim JM, Kim JT, Tarnutzer AA. Video head impulse testing in patients with isolated (hemi)nodular infarction. Front Neurol. 2023 Feb 6;14:1124217. doi: 10.3389/fneur.2023.1124217. PMID: 36814996; PMCID: PMC9939438.
12. Lee SH, Kim JM, Schuknecht B, Tarnutzer AA. Vestibular and Ocular Motor Properties in Lateral Medullary Stroke Critically Depend on the Level of the Medullary Lesion. Front Neurol. 2020 Jun 5;11:390. doi: 10.3389/fneur.2020.00390. PMID: 32655466; PMCID: PMC7325917.
13. Machner B, Erber K, Choi JH, Trillenberg P, Sprenger A, Helmchen C. Usability of the head impulse test in routine clinical practice in the emergency department to differentiate vestibular neuritis from stroke. Eur J Neurol. 2021 May;28(5):1737-1744. doi: 10.1111/ene.14707. Epub 2021 Jan 15. PMID: 33382146.
14. Machner B, Erber K, Choi JH, Sprenger A, Helmchen C, Trillenberg P. A Simple Gain-Based Evaluation of the Video Head Impulse Test Reliably Detects Normal Vestibulo-Ocular Reflex Indicative of Stroke in Patients With Acute Vestibular Syndrome. Front Neurol. 2021 Oct 29;12:741859. doi: 10.3389/fneur.2021.741859. PMID: 34777209; PMCID: PMC8585749.
15. Mantokoudis G, Saber Tehrani AS, Wozniak A, Eibenberger K, Kattah JC, Guede CI, Zee DS, Newman-Toker DE. Impact of artifacts on VOR gain measures by video-oculography in the acute vestibular syndrome. J Vestib Res. 2016 Nov 3;26(4):375-385. doi: 10.3233/VES-160587. PMID: 27814312; PMCID: PMC6054448.
16. Mantokoudis G, Tehrani AS, Wozniak A, Eibenberger K, Kattah JC, Guede CI, Zee DS, Newman-Toker DE. VOR gain by head impulse video-oculography differentiates acute vestibular neuritis from stroke. Otol Neurotol. 2015 Mar;36(3):457-65. doi: 10.1097/MAO.0000000000000638. PMID: 25321888.
17. Michailidou E, Korda A, Wyss T, Bardins S, Schneider E, Morrison M, Wagner F, Caversaccio MD, Mantokoudis G. The value of saccade metrics and VOR gain in detecting a vestibular stroke. J Vestib Res. 2024;34(1):49-61. doi: 10.3233/VES-230083. PMID: 38160379.
18. Morrison M, Korda A, Zamaro E, Wagner F, Caversaccio MD, Sauter TC, Kalla R, Mantokoudis G. Paradigm shift in acute dizziness: is caloric testing obsolete? J Neurol. 2022 Feb;269(2):853-860. doi: 10.1007/s00415-021-10667-7. Epub 2021 Jun 30. PMID: 34191079; PMCID: PMC8782777.
19. Nam GS, Shin HJ, Kang JJ, Lee NR, Oh SY. Clinical Implication of Corrective Saccades in the Video Head Impulse Test for the Diagnosis of Posterior Inferior Cerebellar Artery Infarction. Front Neurol. 2021 Feb 18;12:605040. doi: 10.3389/fneur.2021.605040. PMID: 33679578; PMCID: PMC7930369.
20. Newman-Toker DE, Saber Tehrani AS, Mantokoudis G, Pula JH, Guede CI, Kerber KA, Blitz A, Ying SH, Hsieh YH, Rothman RE, Hanley DF, Zee DS, Kattah JC. Quantitative video-oculography to help diagnose stroke in acute vertigo and dizziness: toward an ECG for the eyes. Stroke. 2013 Apr;44(4):1158-61. doi: 10.1161/STROKEAHA.111.000033. Epub 2013 Mar 5. PMID: 23463752; PMCID: PMC8448203.
21. Nham B, Wang C, Reid N, Calic Z, Kwok BYC, Black DA, Bradshaw A, Halmagyi G, Welgampola MS. Modern vestibular tests can accurately separate stroke and vestibular neuritis. J Neurol. 2023 Apr;270(4):2031-2041. doi: 10.1007/s00415-022-11473-5. Epub 2022 Dec 24. PMID: 36566345.
22. Nham B, Reid N, Bein K, Bradshaw AP, McGarvie LA, Argaet EC, Young AS, Watson SR, Halmagyi GM, Black DA, Welgampola MS. Capturing vertigo in the emergency room: three tools to double the rate of diagnosis. J Neurol. 2022 Jan;269(1):294-306. doi: 10.1007/s00415-021-10627-1. Epub 2021 Aug 16. PMID: 34398269.
23. Olivecrona E, Zborayova K, Barrenäs ML, Salzer J. Comparison Between the Video Head Impulse Test and Caloric Irrigation During Acute Vertigo. Indian J Otolaryngol Head Neck Surg. 2022 Dec;74(Suppl 3):4475-4482. doi: 10.1007/s12070-022-03123-z. Epub 2022 Aug 1. PMID: 36742615; PMCID: PMC9895590.
24. Park JY, Choi JH, Kwon JH, Weon YC, Lee SM, Kim HJ, Choi SY, Oh EH, Kim HA, Lee H, Kim JS, Choi KD. Incidence, characteristics, and neuroanatomical substrates of vestibular symptoms in supratentorial stroke. J Neurol. 2023 Apr;270(4):2174-2183. doi: 10.1007/s00415-023-11566-9. Epub 2023 Jan 12. PMID: 36633670.
25. Siepmann T, Gruener C, Simon E, Sedghi A, Kitzler HH, Pallesen LP, Barlinn J, Reichmann H, Puetz V, Barlinn K. Video-Oculography-Assisted Head Impulse Test and Caloric Testing for Detecting Stroke in Acute Vertigo Patients via Modified HINTS Plus. J Clin Med. 2021 Sep 28;10(19):4471. doi: 10.3390/jcm10194471. PMID: 34640489; PMCID: PMC8509535.
26. Thomas JO, Sharobeam A, Venkat A, Blair C, Ozalp N, Calic Z, Wyllie P, Middleton PM, Welgampola M, Cordato D, Cappelen-Smith C. Video head impulse testing to differentiate vestibular neuritis from posterior circulation stroke in the emergency department: a prospective observational study. BMJ Neurol Open. 2022 May 3;4(1):e000284. doi: 10.1136/bmjno-2022-000284. PMID: 35571585; PMCID: PMC9066478.
27. von Martial R, Leinweber C, Hubert N, Rambold H, Haberl RL, Hubert GJ, Müller-Barna P. Feasibility of Telemedical HINTS (Head Impulse-Nystagmus-Test of Skew) Evaluation in Patients With Acute Dizziness or Vertigo in the Emergency Department of Primary Care Hospitals. Front Neurol. 2022 Feb 11;12:768460. doi: 10.3389/fneur.2021.768460. PMID: 35222226; PMCID: PMC8873087.
28. Zhang Y, Chen S, Zhong Z, Chen L, Wu Y, Zhao G, Liu Y. [Preliminary application of video head impulse test in the diagnosis of vertigo]. Lin Chuang Er Bi Yan Hou Tou Jing Wai Ke Za Zhi. 2015 Jun;29(12):1053-8. Chinese. PMID: 26513989.

*Wrong study design (n = 7)*

1. Koohi N, Mendis S, Lennox A, Whelan D, Kaski D. Video head impulse testing: Pitfalls in neurological patients. J Neurol Sci. 2022 Nov 15;442:120417. doi: 10.1016/j.jns.2022.120417. Epub 2022 Sep 17. PMID: 36209570.
2. Evangelista ASL, Diniz Júnior J, Costa APM, Dourado Júnior MET, Mantello EB. Neurological and vestibular findings in three cases of Multiple Sclerosis. Codas. 2023 Nov 20;35(6):e20210153. doi: 10.1590/2317-1782/20232021153pt. PMID: 37991027; PMCID: PMC10702714.
3. Moreno-Ajona D, Álvarez-Gómez L, Manrique-Huarte R, Rivas E, Martínez-Vila E, Pérez-Fernández N. VEMPs and Dysautonomia Assessment in Definite Cerebellar Ataxia, Neuropathy, Vestibular Areflexia Syndrome (CANVAS): a Case Series Study. Cerebellum. 2021 Oct;20(5):717-723. doi: 10.1007/s12311-019-01061-1. PMID: 31414248.
4. Parker TM, Farrell N, Otero-Millan J, Kheradmand A, McClenney A, Newman-Toker DE. Proof of Concept for an "eyePhone" App to Measure Video Head Impulses. Digit Biomark. 2020 Dec 30;5(1):1-8. doi: 10.1159/000511287. PMID: 33615116; PMCID: PMC7879263.
5. Kim JM, Nam TS, Choi SM, Kim BC, Lee SH. Clinical value of vestibulo-ocular reflex in the differentiation of spinocerebellar ataxias. Sci Rep. 2023 Sep 7;13(1):14783. doi: 10.1038/s41598-023-41924-6. PMID: 37679515; PMCID: PMC10485070.
6. Mahfuz MM, Millar JL, Schubert MC. Repeated video head impulse testing in patients is a stable measure of the passive vestibulo-ocular reflex. J Otol. 2021 Jul;16(3):128-137. doi: 10.1016/j.joto.2020.12.002. Epub 2020 Dec 18. PMID: 34220981; PMCID: PMC8241694.
7. Hermann R, Robert C, Lagadec V, Dupre M, Pelisson D, Froment Tilikete C. Catch-Up Saccades in Vestibular Hypofunction: A Contribution of the Cerebellum? Cerebellum. 2024 Feb;23(1):136-143. doi: 10.1007/s12311-023-01512-w. Epub 2023 Jan 21. Erratum in: Cerebellum. 2024 Feb;23(1):144. doi: 10.1007/s12311-023-01530-8. PMID: 36680705; PMCID: PMC10864466.

*Wrong population (n = 5)*

1. Mantokoudis G, Saber Tehrani AS, Kattah JC, Eibenberger K, Guede CI, Zee DS, Newman-Toker DE. Quantifying the vestibulo-ocular reflex with video-oculography: nature and frequency of artifacts. Audiol Neurootol. 2015;20(1):39-50. doi: 10.1159/000362780. Epub 2014 Dec 9. PMID: 25501133.
2. Hansson A, Salzer J. Normative video head impulse test data in subjects with and without vascular risk factors. Eur Arch Otorhinolaryngol. 2021 Jul;278(7):2619-2624. doi: 10.1007/s00405-020-06332-w. Epub 2020 Sep 10. PMID: 32914256; PMCID: PMC8165054.
3. Leng Y, Zhao Y, Zhou H, Ling X, Wang X, Zhao G, Zhang W. The vestibular and oculomotor dysfunction in Fabry disease: a cohort study in China. Ann Med. 2025 Dec;57(1):2453626. doi: 10.1080/07853890.2025.2453626. Epub 2025 Jan 25. PMID: 39862133; PMCID: PMC11770862.
4. Dupré M, Hermann R, Vidoni L, Quadrio I, Latour P, Subtil F, Froment Tilikete C. Impact of the intronic RFC1 expansion size in CANVAS phenotype: an oculomotor study. J Neurol. 2025 Jun 3;272(6):442. doi: 10.1007/s00415-025-13150-9. PMID: 40461673; PMCID: PMC12134041.
5. Micarelli A, Viziano A, Della-Morte D, Augimeri I, Alessandrini M. Degree of Functional Impairment Associated With Vestibular Hypofunction Among Older Adults With Cognitive Decline. Otol Neurotol. 2018 Jun;39(5):e392-e400. doi: 10.1097/MAO.0000000000001746. PMID: 29547458.

*Wrong publication type (n = 1)*

1. Siepmann T, Gruener C, Simon E, Winzer S, Sedghi A, Pallesen L‐P, Barlinn J, Puetz V, Barlinn K. Caloric testing combined with assessment of nystagmus, skew and hearing loss outperforms conventional and videooculography supported hints-plus in detecting stroke presenting as acute vestibular syndrome. European stroke journal, 2021, 6(1 SUPPL), 177‐178. doi: 10.1177/23969873211034932.

*Wrong outcome (n = 1)*

1. Misale P, Hassannia F, Dabiri S, Brandstaetter T, Rutka J. Post-traumatic peripheral vestibular disorders (excluding positional vertigo) in workers following head injury. Sci Rep. 2021 Dec 6;11(1):23436. doi: 10.1038/s41598-021-02987-5. PMID: 34873257; PMCID: PMC8648866.

*Wrong intervention (n = 1)*

1. Asquier-Khati A, Mauhin W, Michel G, Gendre A, Durant C, Lavigne C, Maillard H, Lacombe D, Willems M, Lidove O, Masseau A. Cochleovestibular involvement in patients with Fabry disease: data from the multicenter cohort FFABRY. Eur Arch Otorhinolaryngol. 2022 Mar;279(3):1639-1644. doi: 10.1007/s00405-021-07173-x. Epub 2021 Nov 26. PMID: 34825971.

**eMethods 3.** Quality of studies included

|  | **Q1** | **Q2** | **Q3** | **Q4** | **Q5** | **Q6** | **Q7** | **Q8** | **Q9** |
| --- | --- | --- | --- | --- | --- | --- | --- | --- | --- |
| Alshehri et al. 2016^19^ | ⚠️ | ⚠️ | ❌ | ✅ | ✅ | ✅ | ✅ | ✅ | ⚠️ |
| Anagnostou et al. 2019^20^ | ✅ | ⚠️ | ❌ | ❌ | ✅ | ✅ | ✅ | ✅ | ✅ |
| Ariello et al. 2025^59^ | ✅ | ⚠️ | ⚠️ | ✅ | ⚠️ | ✅ | ✅ | ✅ | ⚠️ |
| Aydin Canturk et al. 2023^22^ | ✅ | ⚠️ | ✅ | ❌ | ✅ | ✅ | ⚠️ | ✅ | ❌ |
| Berkiten et al. 2023^21^ | ✅ | ⚠️ | ✅ | ✅ | ✅ | ✅ | ✅ | ✅ | ✅ |
| Borsche et al. 2023^23^ | ✅ | ✅ | ❌ | ✅ | ✅ | ✅ | ✅ | ✅ | ✅ |
| Bosmans et al. 2024^24^ | ✅ | ✅ | ⚠️ | ✅ | ✅ | ✅ | ✅ | ✅ | ✅ |
| Bremova et al. 2016^25^ | ✅ | ✅ | ❌ | ✅ | ✅ | ✅ | ✅ | ✅ | ✅ |
| Choi et al. 2024^26^ | ✅ | ✅ | ❌ | ✅ | ✅ | ✅ | ✅ | ✅ | ✅ |
| Dankova et al. 2021^27^ | ✅ | ✅ | ⚠️ | ✅ | ✅ | ✅ | ✅ | ✅ | ✅ |
| Demir et al. 2023^28^ | ⚠️ | ⚠️ | ❌ | ✅ | ✅ | ✅ | ✅ | ✅ | ✅ |
| Egilmez et al. 2022^29^ | ✅ | ✅ | ❌ | ✅ | ⚠️ | ✅ | ✅ | ✅ | ✅ |
| Elyoseph et al. 2024^30^ | ✅ | ✅ | ❌ | ✅ | ✅ | ✅ | ✅ | ✅ | ✅ |
| Feller et al. 2024^63^ | ✅ | ✅ | ❌ | ✅ | ✅ | ❌ | ✅ | ✅ | ✅ |
| Fernandez-Rueda et al. 2023^31^ | ✅ | ✅ | ❌ | ✅ | ✅ | ✅ | ✅ | ⚠️ | ✅ |
| Ferri et al. 2025a^60^ | ✅ | ⚠️ | ⚠️ | ✅ | ✅ | ✅ | ✅ | ✅ | ✅ |
| Ferri et al. 2025b^62^ | ✅ | ✅ | ❌ | ✅ | ✅ | ✅ | ✅ | ✅ | ✅ |
| Grove et al. 2022a^33^ | ✅ | ✅ | ❌ | ✅ | ✅ | ✅ | ✅ | ✅ | ✅ |
| Grove et al. 2022b^34^ | ✅ | ✅ | ✅ | ✅ | ✅ | ✅ | ✅ | ✅ | ✅ |
| Hawkins et al. 2022^35^ | ✅ | ✅ | ✅ | ✅ | ✅ | ✅ | ✅ | ✅ | ✅ |
| Heravian Shandiz et al. 2021^36^ | ❌ | ❌ | ❌ | ✅ | ❌ | ✅ | ⚠️ | ✅ | ✅ |
| Hong et al. 2022^37^ | ✅ | ✅ | ✅ | ✅ | ✅ | ✅ | ✅ | ✅ | ✅ |
| Hougaard et al. 2019^38^ | ✅ | ✅ | ❌ | ✅ | ✅ | ✅ | ✅ | ⚠️ | ✅ |
| Kim et al. 2022^39^ | ✅ | ✅ | ❌ | ✅ | ⚠️ | ✅ | ✅ | ✅ | ✅ |
| Kim et al. 2023^40^ | ✅ | ✅ | ❌ | ✅ | ✅ | ✅ | ✅ | ✅ | ✅ |
| Kim et al. 2025a^65^ | ✅ | ✅ | ✅ | ✅ | ✅ | ✅ | ✅ | ✅ | ✅ |
| Kim et al. 2025b^61^ | ❌ | ❌ | ✅ | ✅ | ✅ | ✅ | ✅ | ✅ | ✅ |
| Le et al. 2020^41^ | ✅ | ✅ | ❌ | ✅ | ❌ | ✅ | ✅ | ✅ | ✅ |
| Lee et al. 2020^42^ | ❌ | ✅ | ❌ | ✅ | ✅ | ✅ | ✅ | ✅ | ✅ |
| Lemos et al. 2021^43^ | ✅ | ✅ | ⚠️ | ✅ | ✅ | ✅ | ✅ | ✅ | ✅ |
| Luis et al. 2016^44^ | ✅ | ⚠️ | ❌ | ⚠️ | ✅ | ✅ | ✅ | ✅ | ✅ |
| Lv et al. 2017^45^ | ✅ | ✅ | ⚠️ | ✅ | ✅ | ✅ | ✅ | ✅ | ✅ |
| Millar et al. 2022^46^ | ✅ | ✅ | ❌ | ❌ | ✅ | ✅ | ✅ | ✅ | ✅ |
| Oron et al. 2020^47^ | ✅ | ⚠️ | ❌ | ✅ | ✅ | ✅ | ⚠️ | ⚠️ | ✅ |
| Pavlovic et al. 2017^48^ | ❌ | ❌ | ❌ | ✅ | ✅ | ✅ | ✅ | ✅ | ✅ |
| Scarpa et al. 2020^49^ | ✅ | ✅ | ❌ | ✅ | ✅ | ✅ | ✅ | ✅ | ✅ |
| Sonkaya et al. 2024^66^ | ✅ | ✅ | ❌ | ✅ | ✅ | ✅ | ✅ | ✅ | ✅ |
| Surmeli et al. 2020a^51^ | ❌ | ✅ | ❌ | ✅ | ✅ | ✅ | ✅ | ✅ | ✅ |
| Surmeli et al. 2020b^50^ | ✅ | ✅ | ❌ | ✅ | ✅ | ✅ | ✅ | ✅ | ✅ |
| Takeda et al. 2018^52^ | ✅ | ✅ | ❌ | ✅ | ❌ | ✅ | ✅ | ⚠️ | ✅ |
| Taylor et al. 2022^53^ | ✅ | ✅ | ✅ | ✅ | ✅ | ✅ | ✅ | ✅ | ✅ |
| Tramontano et al. 2024a^54^ | ✅ | ✅ | ❌ | ✅ | ✅ | ✅ | ✅ | ✅ | ✅ |
| Tramontano et al. 2024b^64^ | ✅ | ✅ | ❌ | ✅ | ✅ | ✅ | ✅ | ✅ | ✅ |
| Wagner et al. 2019^56^ | ⚠️ | ⚠️ | ❌ | ✅ | ✅ | ✅ | ⚠️ | ⚠️ | ✅ |
| Wagner et al. 2022^55^ | ❌ | ✅ | ⚠️ | ✅ | ✅ | ✅ | ✅ | ✅ | ✅ |
| Woo et al. 2024^57^ | ⚠️ | ✅ | ✅ | ✅ | ✅ | ✅ | ✅ | ✅ | ✅ |
| Yacovino et al. 2019^58^ | ❌ | ⚠️ | ❌ | ✅ | ❌ | ✅ | ✅ | ❌ | ✅ |
